# Supplementary material for: Inflammatory factors and risk of lung adenocarcinoma: a Mendelian randomization study mediated by blood metabolites
Source: Front Endocrinol (Lausanne). 2024 Aug 27;15:1446863. doi: 10.3389/fendo.2024.1446863 (PMC11384989; doi:10.3389/fendo.2024.1446863)
Supplement: Supplementary file 1 [file DataSheet1.zip › supplementary files/S2/GCST90200982/sensitivity-analysis.pdf]

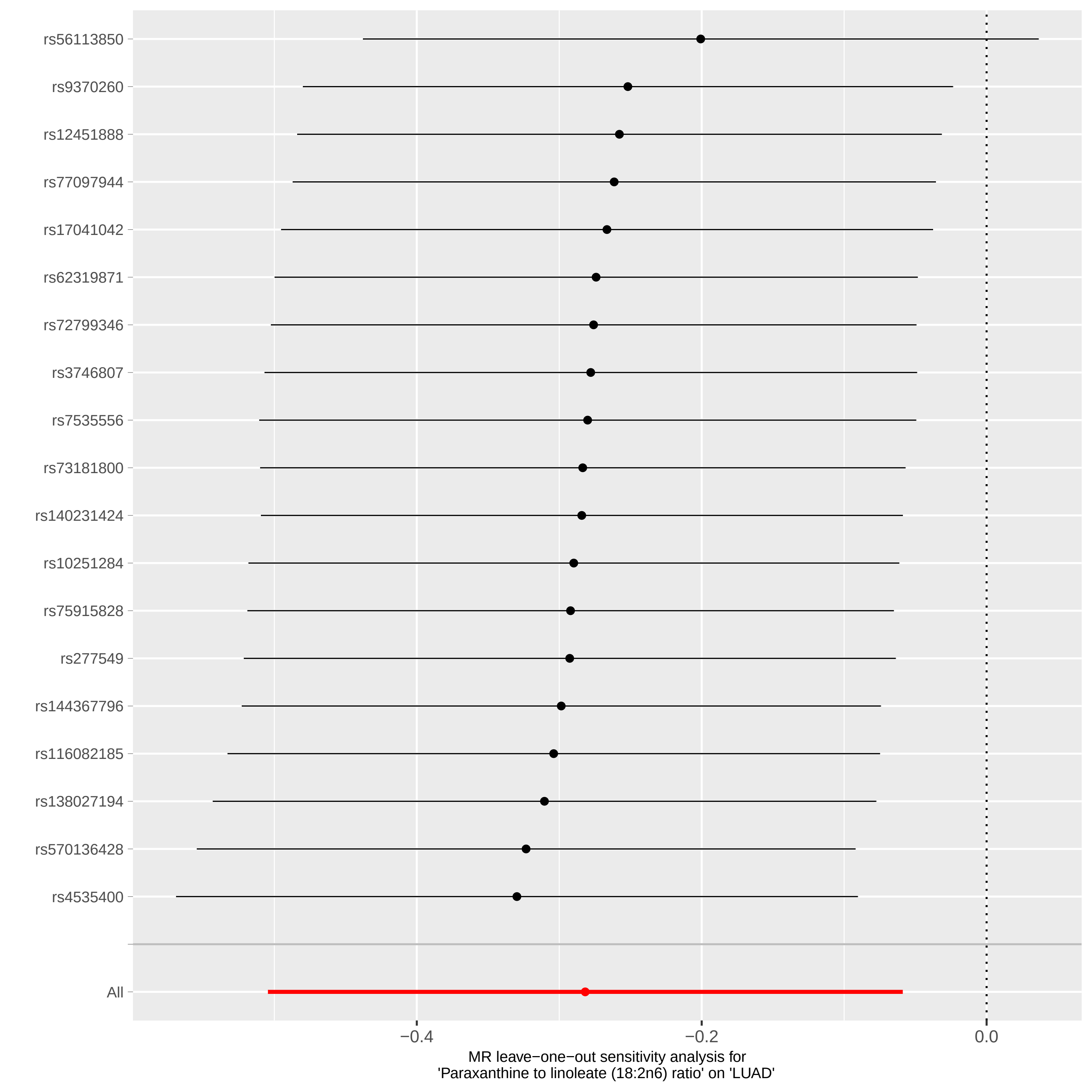
rs56113850
rs9370260
rs12451888
rs77097944
rs17041042
rs62319871
rs72799346
rs3746807
rs7535556
rs73181800
rs140231424
rs10251284
rs75915828
rs277549
rs144367796
rs116082185
rs138027194
rs570136428
rs4535400
All
−0.4
−0.2
0.0
MR leave−one−out sensitivity analysis for
'Paraxanthine to linoleate (18:2n6) ratio' on 'LUAD'
